# Education in Time: Cohort Differences in Educational Attainment in African-American Twins

**DOI:** 10.1371/journal.pone.0007664

**Published:** 2009-10-30

**Authors:** Sarah L. Szanton, Brandon Johnson, Roland J. Thorpe, Keith Whitfield

**Affiliations:** 1 Johns Hopkins School of Nursing, Johns Hopkins University, Baltimore, Maryland, United States of America; 2 Center on Aging and Health, Johns Hopkins Medical Institutions, Baltimore, Maryland, United States of America; 3 Hopkins Center for Health Disparities Solutions, Johns Hopkins Bloomberg School of Public Health, Baltimore, Maryland, United States of America; 4 Department of Health Policy and Management, Johns Hopkins Bloomberg School of Public Health, Baltimore, Maryland, United States of America; 5 Department of Psychology and Neuroscience, Duke University, Durham, North Carolina, United States of America; Aga Khan University Hospital, Pakistan

## Abstract

**Objectives:**

Educational opportunities for African-Americans expanded throughout the 20^th^ century. Twin pairs are an informative population in which to examine changes in educational attainment because each twin has the same parents and childhood socioeconomic status. We hypothesized that correlation in educational attainment of older twin pairs would be higher compared to younger twin pairs reflecting changes in educational access over time and potentially reflecting a “ceiling effect” associated with Jim Crow laws and discrimination.

**Methodology and Principal Findings:**

We used data from 211 same-sex twin pairs (98 identical, 113 fraternal) in the Carolina African-American Twin Study of Aging who were identified through birth records. Participants completed an in-person interview. The twins were predominantly female (61%), with a mean age of 50 years (SD = 0.5). We found that older age groups had a stronger intra-twin correlation of attained educational level. Further analysis across strata revealed a trend across zygosity, with identical twins demonstrating more similar educational attainment levels than did their fraternal twin counterparts, suggesting a genetic influence.

**Discussion:**

These findings suggest that as educational opportunities broadened in the 20th century, African-Americans gained access to educational opportunities that better matched their individual abilities.

## Introduction

Educational attainment is an enduring predictor of adult health. Higher educational attainment is associated with decreased mortality [1]–[3], decreased disability [4], [5], and decreased cardiovascular disease [6]. Link and Phelan have posited [7] the ability of those with higher education to take advantage of resources, attain fulfilling jobs, respond to new health information, and gain access to health care. However, the connection between higher levels of education and increased cognitive functioning is another possible pathway by which increased education can lead to better health [8].

Educational attainment was historically severely limited among African-Americans, particularly in the South. By 1831, it was illegal for slaves to be taught to read or write in all of the Southern states [9]. Restrictions on education gradually lessened but remained during the Jim Crow era. During that period, schools were racially segregated, and the quality of black schools was markedly inferior to that of white schools [10].

Despite such legalized barriers to education, educational possibilities for African-Americans in the South increased markedly over the course of the 20^th^ century. This time-period effect engendered dissimilar educational opportunities, on average, for different age cohorts now alive [11]. For example, African-Americans who are currently in their 80s experienced a different educational climate when they were school-aged than did those who are now in their 40s.

Because education is an important determinant of an adult's access to resources and health, it is hard to overstate the importance of this transformation in opportunities. Social policies such as desegregation and civil rights expansion are likely responsible for some of the increases in 20^th^ century African Americans' educational attainment, but their influence has not been examined in detail.

Twin pairs are a potentially informative population in which to examine changes in educational attainment because each pair has the same parents and childhood socioeconomic status, two known determinants of educational attainment. Because genetic variance is likely constant over time, then an increase in phenotypic variance is likely attributable to increases in environmental variance. We therefore hypothesized that twins born later in the 20^th^ century would be less similar to each other in educational attainment than those born earlier in the 20^th^ century and that heritability estimates would therefore decrease over time. We hypothesized that the educational attainment of older twin pairs would be more highly correlated than that of younger twin pairs because of these changes in educational access in the South over time, potentially reflecting a “ceiling effect” associated with the Jim Crow laws and discrimination. The aim of this study was to test that hypothesis.

Twin studies have long been used to compare the intra-twin similarity of identical (monozygotic) twins to that of fraternal (dizygotic) twins, as a means of estimating genetic and environmental effects. While our focus here was not exclusively on estimating genetic effects, our study design allowed us to identify the relative levels to which genetic and environmental factors contributed to educational attainment in African-Americans across age cohorts. The extent to which genetic factors influenced educational attainment provided insight into genes that affect characteristics such as personality, rather than the actual ability to attain an education.

## Methods

### Ethics Statement

All participants gave written informed consent, and the study was approved by the Institutional Review Boards of the University of North Carolina Chapel Hill and Pennsylvania State University.

### Study Population

Data for these analyses were obtained from the Carolina African American Twin Study of Aging (CAATSA) [12]. The CAATSA was designed to examine the health status, cognitive functioning, and physical and psychosocial functioning of adult African-American twins. This population-based sample of participants was identified from birth records for the years 1913-1975 from 23 vital statistics offices in North Carolina counties. Birth records were then entered into a computerized database of twin births. After the records were computerized, potential participants were located through voter registries and telephone white page searches.

Assessments of educational attainment and age were completed in person by a trained interviewer. Participants were enrolled between 1999 and 2003. Additional information regarding the CAATSA study design can be found elsewhere [12]. The CAATSA sample consisted of 285 pairs of twins. We dropped opposite sex fraternal twins (N = 62 pairs) to eliminate the variability in gendered access to education. We also eliminated the three twin pairs who were not raised together, (separated prior to age 13) based on other twin research with twins' socioeconomic opportunity [13]. Finally, we dropped those who had missing data re years of education attained (N = 8). The final sample was 211 same sex twins, 99 identical and 113 fraternal.

#### Ascertainment of age

The sample was identified through birth records that included a birth date for the twin pairs. The trained interviewer also verbally confirmed the age of each participant in person.

#### Ascertainment of education

The interviewer asked the participants for their highest year of education attained.

### Statistical Analysis

Educational attainment was first examined as a function of age, and the overall sample was then divided into identical and fraternal and twins. Correlations between the two members of each twin pair were then assessed according to age group (by decades) for both identical and fraternal twins. Pearson's correlations were calculated for the intra-twin correlations for each age group and according to twin type. All analyses were done in STATA, version 10.

## Results

The 422 adults in our analytic sample had a mean age of 50 years (SD = 14.6), with a range of 40–79 years Overall, their mean educational attainment was 13.3 years (SD = 2.90), with a range of 4 to 30 years.

As expected, the mean educational attainment progressively increased with decreasing age of the groups: It increased from 5.67 for the 80–89 age group to 12.3, 12.6, 13.3, 13.6, 13.9, and 14.0 in the 70–79, 60–69, 50–59, 40–49, 30–39, and 20–29 age groups, respectively. Our main finding was that identical twins showed a correlation with their own twins in terms of educational attainment at the level of 0.93, 0.83, 0.89, and 0.70 for the 70–79, 60–69, 50–59, and 40–49 age groups, respectively; in contrast, fraternal twins showed correlations of 0.65, 0.54, 0.50, and 0.35, respectively, for the same age groups (see Table 1). An estimate of the contribution of genetic influences can be calculated using the formula: *h^2^* = 2(rMZ-rDZ), where MZ indicates identical twins and DZ fraternal twins (c.f. Plomin[14]. Using this straightforward formula, the heritability values were 0.74, 0.56, 0.80, and 0.78 for the 70–79, 60–69, 50–59, and 40–49 age groups, respectively. The remainder of the individual variability was considered to be due to environmental factors.

**Table 1:** Years of education by age- group with identical and fraternal intra-twin correlations.

Age group	Number of twin pairs	Mean education (STD, range)	*Correlation to twin's education (identical)	*Correlation to twin's education (fraternal)
40–49	58	13.5 (2.8, 7–21)	0.70	0.35
50–59	55	13.5 (3.4, 6–30)	0.89	0.50
60–69	20	12.8 (4.0, 7–21)	0.83	0.54
70–79	14	11.8 (4.1, 4–21)	0.93	0.65

* Pearson correlations between twin pairs on years of education attained.

## Discussion

As we had hypothesized, older African-American twins showed an almost complete correlation with their respective twins in terms of educational attainment, likely reflecting the ceiling effect of Jim Crow laws that limited African-American education. For the groups who were born progressively later and later in the 20^th^ century, the correlation between twins became smaller and smaller, perhaps as a result of the lessening of these laws and advances in civil rights that allowed each twin to pursue the amount of education relevant to his/her own motivations, ability, personal interests, and career plans.

We had also hypothesized that there would be a genetic influence on educational attainment. As predicted, we found that for every age group, the identical twin pairs had a greater correlation than did the comparable fraternal twin pairs. The results suggest that there is a substantial education-related genetic component affecting each age group. Several twin studies have examined differences in education between twins. Unpublished data from the Minnesota Twin Registry yielded heritabilities of 0.3 to 0.4; the participants in that study were predominately Caucasian. Other studies have analyzed intra-twin differences in education in relationship to divergent adult health [13], [15]. Such an analysis in our population did not generate significant results because of the high correlations in educational attainment. To our knowledge, ours is the only study to examine time-period effects on the correlation of educational attainment between twins.

The pattern we uncovered in decreasing intra-twin pair correlation in educational attainment was not statistically significant but we believe the pattern is noteworthy. Because educational attainment is usually achieved in the first three decades of life, education is a strong summary measure of childhood opportunities. In this group of study participants, such childhood opportunities changed dramatically over the course of the twentieth century. In this data, we have a unique exemplar of 422 African American twins spanning educational years of 1930 to 1980. This rich but moderate sized data set allows us to control for socioeconomic status and parental education by design but not to have enough twins to find statistical significance.

It should be noted that our analysis measured educational quantity, and not quality. A particular number of years of attainment does not necessarily confer the same benefit on all individuals in terms or cognition, physical health, or socioeconomic status because of differences in educational investment from state to state and county to county [16], [17]. A further complicating factor was identified by Whitfield and Wiggins (2003), who demonstrated that there were positive influences on healthy cognitive functioning of attending both segregated and desegregated schools.

In conclusion, our current findings support the concept that as educational opportunities broadened in the 20th century, African-Americans gained access to educational opportunities that matched their individual interests. The data presented here included twins as young as 40. These individuals likely began their formal educational experiences around 1970. It will be important to determine whether the trend toward increasing difference that we identified will continue and to seek explanations for these differences. For example, does the difference in twin pairs' educational attainment in the 1970s, 1980s, and 1990s have more to do with family preferences for individualilty and socioeconomic status (SES) than with civil rights issues? Does the impact of genes become more or less important as a result of environmental factors such as school busing or changes in technology that drive a need for more education? The answers to these research questions will have important implications for health and society.
